# Heat exposure and cardiovascular risk factors among migrant and seasonal farmworkers in the United States: data from the National Agricultural Workers Survey (1999–2020)

**DOI:** 10.1038/s41370-026-00919-x

**Published:** 2026-05-30

**Authors:** Lisbeth Iglesias-Rios, Yike Liu, Marie S. O’Neill, Alexis J. Handal

**Affiliations:** 1 Department of Epidemiology, University of Michigan School of Public Health, Ann Arbor, MI USA; 2 Departments of Epidemiology and Environmental Health Sciences, University of Michigan School of Public Health, Ann Arbor, MI USA

**Keywords:** farmworkers, heat, cardiovascular disease, diabetes, hypertension, occupational health

## Abstract

**Background:**

The United States (U.S.) currently lacks a Federal occupational heat standard and hot weather threatens the safety and health of farmworkers who often work under extreme heat conditions. Chronic disease and related risk factors can enhance worker susceptibility, yet prior studies on heat vulnerability in farmworkers used small samples over short time periods, leaving knowledge gaps about the scope of the problem.

**Objective:**

To describe temperature exposures and characteristics, including cardiovascular disease (CVD) and risk factors (diabetes, high blood pressure, elevated cholesterol), of a U.S. national sample of farmworkers over a 21-year period.

**Methods:**

We obtained demographic and health data on farmworkers from the U.S. National Agricultural Workers Survey (NAWS) and publicly available temperature data from 1999 to 2020. We aggregated temperature datasets to six NAWS regions and calculated average daily mean temperature days above four absolute thresholds of 32.2–40.6 Celsius (°C) and seasonal average summertime temperatures to represent potential exposure of the survey population. We used joinpoint regression to describe temporal patterns in temperatures and the prevalence of CVD outcomes within the repeated cross-sectional NAWS data. Multivariate logistic regression analysis was used to explore patterns in CVD outcomes across farmworker characteristics.

**Results:**

Farmworkers (*n* = 44,388) were predominantly Hispanic (81%) and male (73%) with 33% below the poverty level and 40% receiving benefits from needs-based government programs. Seasonal temperatures experienced overall increasing trends, and all regions experienced days over heat thresholds. Prevalence of diabetes, high blood pressure, and CVD generally increased over the study period, with some diminishing trends for diabetes and high blood pressure after 2017.

**Significance:**

Increasing prevalence of CVD and related risk factors, combined with the aging workforce and other social vulnerabilities, support targeted actions to mitigate heat-related health risks and ensure safety and health provisions for this essential workforce.

**Impact Statement:**

- Elevated temperatures pose a risk of morbidity and mortality for farmworkers and outdoor workers in general. We present evidence from a nationwide survey that U.S. farmworkers, while generally younger and healthier than the population as a whole, experience potential vulnerability to heat as their prevalence of cardiovascular disease, average age and risk factors have risen nationally over 21 years. These results highlight the need to pass and implement heat standards and policies to ensure the safety and health of this essential workforce, in light of the absence of such standards in all but five states.

## Introduction

Extreme heat events have become more frequent in the United States (U.S.) in the last decades due to elevated global temperature and climate change [1–4]. From 2004-2018, approximately 700 deaths directly attributed to heat were estimated to occur per year in the total population in the U.S [5]. From January 1, 2017, to December 31, 2022, 1054 heat-related injuries, illnesses, and fatalities in various occupations, including agriculture, were reported to and investigated by the U.S. Occupational Safety and Health Administration (OSHA), including 625 heat-related hospitalizations, 211 heat-related fatalities, and 218 heat-related injuries and illnesses that did not result in hospitalization [6]. These numbers likely underestimate the total burden of heat on mortality, since heat is a contributing factor in deaths from cardiovascular, renal, respiratory, and other causes even if heat-related causes are not reported on the death certificate or other records [7]. People who work outdoors or indoors in settings where temperatures are elevated can be particularly at risk during hot weather conditions [2, 8]. Currently, no federal heat standards protect workers in the U.S., but OSHA proposed in 2024 to issue new heat standards for indoor and outdoor workers [7].

### Heat is an important environmental justice issue for farmworkers

Extreme heat events and heat-related deaths disproportionately impact individuals from minoritized communities in rural and urban areas [2, 9]. Historical policies in the United States, including use of migrant labor and exclusion of farmworkers from protections, have contributed to environmental injustices and precarious work conditions for farmworkers [10]. Climate change-related exposures can additionally pose challenges to safe, healthy, and decent working conditions that protect worker wellbeing and comfort. The Environmental Justice Principles, which were established in 1991 at the National People of Color Environmental Leadership Summit, assert: “the fundamental right of all workers to a safe and healthy work environment without being forced to choose between an unsafe livelihood and unemployment.” [11] Heat exposure is a critical issue for farmworkers, as heat stress and heat related illness are a major concern for this vulnerable population overrepresented by Latinos in the U.S. who are exempted from labor and social protections, and at high risk for heat-related adverse health outcomes [9, 10, 12].

### Heat exposure in farmworkers and cardiovascular disease

Heat exposure has been a key issue for occupational health and safety in the agricultural workforce. A bill named after *Asunción Valdivia* who died in 2004 from heat stroke after picking grapes for ten hours straight in 40.6 °C degree temperatures was introduced in the 117th Congress in 2021–2022, requiring OSHA to create a national heat standard. However, the bill died in January 2023 and the country continues to lack federal regulations on heat standards for outdoor workers [13]. Agricultural workers experience a greater incidence of heat-related illness (HRI) and death compared to workers in other industries [12, 14–17]. Prolonged exposure to high temperatures, humidity, and direct sunlight combined with the effects of high metabolic heat production associated with occupational tasks, high ambient and radiant heat, and low air flow, add to human heat strain [18]. For farmworkers specifically, productivity has been shown to decrease with heat stress [19]. Use of protective clothing and equipment may further exacerbate workers’ risk by preventing the body from cooling, while personal factors such as hydration, medication use, pregnancy, and overall health status also contribute to heat-related health outcomes [20, 21].

Research shows that all-cause cardiovascular illness is a primary cause of death during heatwaves [18, 22, 23]. While the underlying mechanisms behind health effects of heatwaves are not completely understood, many pathophysiological responses associated with extremely high temperatures have been postulated to be responsible for effects on cardiovascular health [24, 25]. For example, exposure to extremely high temperatures could induce elevated heart rate, affect blood viscosity and plasma cholesterol levels, reduce cerebral perfusion, attenuate vasoconstrictor responsiveness, alter fluid and electrolytic balance, and cause airway hyper-responsiveness, which could trigger cardiovascular symptoms [24, 26].

Farmworkers are known to have a high prevalence of pre-existing chronic illnesses such as diabetes, hypertension, elevated cholesterol, and cardiovascular disease (CVD) and these conditions can confer vulnerability to heat and heatwaves [15, 16, 27–29]. A case-series study by OSHA (OSHA, 2011–2016) identified 64 workers who experienced 66 total episodes of HRI; among these workers, 24 died. Those workers who died of HRI, compared to those who survived, had a higher prevalence of obesity, diabetes, hypertension, and CVD (74% vs 33%) [30]. In part because of the lack of a federal heat standard, few safety inspections for heat-related prevention and management strategies occur on U.S. farms. However, California, Washington, Colorado, Oregon, and Maryland, which do have standards, all require preventive measures that are triggered by high heat [6, 31].

### Motivation and aims of present study

Acute heat exposure disproportionately leads to heat exhaustion and HRI among individuals with CVD or elevated risk for developing CVD, so a better understanding of the distribution of these conditions in the farmworker population which experiences high levels of social marginalization and occupational precarity is needed. Given the threat of heat exposure to worker’s health, this paper aimed to describe hot temperature exposures and CVD risk factors and CVD in hired farmworkers, leveraging national data.

## Methods

### National agricultural workers survey (NAWS)

After securing ethical approval and permission to access data, we obtained data from 1999 to 2020 from the NAWS, a complex multistage, employment-based survey conducted by the U.S. Department of Labor that includes migrant and seasonal farmworkers [32]. The NAWS is implemented during U.S. government fiscal years; as an example, the 2020 fiscal year is defined as October 1, 2019 through September 30, 2020. However, we used calendar years to match NAWS with temperature data, which is reported in calendar years. NAWS does not include farmworkers with H-2A temporary work non-immigrant visas or employees not engaged in crop-related work. The primary purpose of the NAWS is to monitor and assess the terms and conditions of agricultural employment and characteristics of hired farmworkers. The NAWS is designed to address the information needs of various U.S. federal agencies concerned with occupational injury and health surveillance and that oversee farmworker programs. These stakeholders include the Employment and Training Administration at the Department of Labor, the Environmental Protection Agency, and the Department of Health and Human Services. The NAWS differs from many federal worker surveys in that: 1) it is an establishment survey (workers are sampled at their workplaces); 2) only currently employed persons are sampled; and 3) data is collected through face-to-face interviews with farmworkers [32].

The use of an employer-based sample rather than a household-based sample increases the likelihood that migrant workers will be interviewed in the NAWS. Details of NAWS methodology are published elsewhere [32]. Briefly, NAWS has seven sampling levels: cycle, region, farm labor area (primary sampling unit), county, ZIP code, employer, and farmworker. The selection of regions and participant farms is dependent on the amount of farm labor in a region during a particular cycle. The NAWS collects data in 12 regions, six of which are available for public use: East, Southeast, Midwest, Southwest, Northwest, and California. In every cycle, within every region, a random sample of farm labor areas (FLAs) is selected from a roster constructed from the universe of FLAs using probabilities proportional to the size of a given FLA’s seasonal farm labor expenditures. Similarly, counties are randomly selected in each FLA using farm labor expenditures. Within each county, ZIP Code regions are randomly ordered. In the penultimate sampling stage, a simple random sample of agricultural employers is selected from a list of employers. Once the sample of employers is drawn, interviewers contact the selected growers or contractors to obtain access to the work site. The survey collects information on wages and working conditions, legal status, occupational health, and recruitment practices. Workers are interviewed alone to protect their privacy and informed of the purposes of the information collection as well as the safeguards to protect its confidentiality. Additionally, farmworker respondents are protected by the Employment and Training Agency’s System of Records for the NAWS, which was established under the Privacy Act (5 USC 552a). At the conclusion of the survey, all records of the names and addresses are destroyed [33].

### Farmworker employment characteristics and self-reported CVD risk factors and CVD

We examined farmworkers’ self-reported CVD risk factors including diabetes, high blood pressure, elevated cholesterol, and CVD. Diabetes, high blood pressure, and elevated cholesterol are part of the ‘metabolic syndrome’ that can be precursors of CVD [34], and, as noted previously, these chronic conditions themselves may mark particular susceptibility to heat [35]. These conditions were assessed by asking workers, “Have you ever been told by a doctor or nurse (health practitioner) that you have the following condition?” Response options were Yes/No.

We also selected employment characteristics available in the NAWS that may affect heat exposure in farmworkers or be important determinants of the disease outcomes. The selection of these variables was based on our previous research in precarious work and the literature [10, 36, 37], and included: type of farmworker (migrant or not migrant); income; working hours; type of payment (hourly wage, piece-rate, combination hourly wage and piece-rate, salary or other); type of employer (grower or contractor); worker’s health insurance (yes/no); spouse health insurance (yes/no); number of working days and weeks in the past 12 months; type of crop at the time of the interview; benefits from contributions-based federal programs (yes/no); benefits from needs**-**based federal programs (yes/no). Contribution-based benefits in NAWS include disability insurance, unemployment insurance, social security, and veterans’ pay. Need-based benefits include financial assistance through programs such as Temporary Assistance for Needy Families (TANF), general assistance or welfare, and publicly provided housing or medical and nutritional assistance such as Medicaid, Special Supplemental Nutrition Program for Women, Infants and Children (WIC), and Supplemental Nutrition Assistance Program (SNAP).

### Methodological comment

Evidence on associations between chronic heat exposure as a contributing factor to the development of CVD, and acute exposure and the exacerbation of cardiovascular symptoms within the large NAWS national sample would be informative for prevention. However, the NAWS survey does not follow workers longitudinally, but is rather a repeated random sample as noted above, which precludes reliable assessment of chronic exposure; additionally, the time of diagnosis with the chronic health conditions described above is not available, nor are short-term reports of symptoms, or clinical measures. Therefore, the contribution of the present analysis is to provide summer season heat exposure estimates matched to the NAWS sample in descriptive epidemiology, to evaluate and illustrate potential exposures and trends, rather than to assess associations between heat exposure and health outcomes, given the above limitations.

### Temperature metrics

We used data on historical temperatures from the U.S. Centers for Disease Control and Prevention (CDC) National Environmental Public Health Tracking Network [38] and the National Oceanic and Atmospheric Administration (NOAA) [39] statewide time series from 1999 to 2020 to match the period for which data from the NAWS were available. The data were originally provided in Fahrenheit and converted to Celsius values using the standard formula: °C = (°F − 32) × 5/9.

#### National environmental public health tracking network, CDC

We used the query tools to download temperature data from the National Environmental Public Health Tracking Network. The data query included: ‘heat and heat-related illness-historical temperature & heat index, annual number of extreme heat days (full year), all counties.’ [38] The heat metric selected was daily maximum temperature and absolute thresholds from 32.2 to 40.6 °C (90–105 °F) for the years 1999–2020. These daily heat metrics were determined for each county and census tract collected for May to September values only. These CDC measures are derived from estimates of air temperature (K) at 2 m above the surface, specific humidity (kg/kg) at 2 meters above the surface, and surface pressure (Pa) from Forcing File A of Phase 2 of the North American Land Data Assimilation System (NLDAS-2). Maximum daily temperature was determined for each block group (~600 to 3000 people) by identifying the maximum hourly value for each day. The CDC uses census block group population as weights, and population-weighted averages by U.S. county and census tract were calculated. Annual values for extreme heat events represent the total number of days in which an extreme heat event was ongoing. The daily heat metrics were determined for each county and census tract and extreme heat days were classified as days above absolute threshold (32.2, 35.0, 37.8, and 40.6°C) values. We obtained the county-level absolute threshold temperatures data, to examine regional patterns of extreme temperature exposure. For each year from 1999 to 2020, we calculated the total number of days with maximum temperatures exceeding each threshold for individual counties. These county-level totals were then aggregated to represent NAWS regions (California, Southeast, Southwest, East, Midwest, and Northwest) and provide regional summaries.

#### National centers for environmental information (NOAA)

NOAA provides real-time analysis of monthly temperature in the US with the purpose to study climate variability. For each of 344 climate divisions, monthly station temperature and precipitation values are computed from the daily observations. The divisional values are weighted by area to compute statewide values and the statewide values are weighted by area to compute regional values [40]. Temperature data from NOAA was queried as follows: ‘statewide time series, monthly (January-December) maximum temperature from 1999 to 2000. NOAA statewide data is provided by the US Climate Divisional database [41], originally developed for monitoring of drought, temperature, precipitation, and heating/cooling degree day values [39]. We aggregated temperature data from 1999 to 2020 for each U.S. state into the 6 NAWS-defined regions and categorized it by seasons for each year: as December, January, and February for winter; March, April, and May for spring; June, July, and August for summer; and September, October, and November for fall. This was done to mirror the NAWS time frame for employment data collected and seasonal interviewing cycles. For this paper, we focus on the summer season, the months with the highest temperatures of the year, and we also extracted summer minimum temperatures, since nighttime cooling can affect ability to recover from warm daytime temperatures.

### Statistical analysis

All analyses accounted for the multistage complex sampling design of NAWS using weights, cluster, and strata statements. The analysis was restricted to adult participants (≥18 years old) in NAWS given the focus on chronic disease. We described sociodemographic, employment characteristics, CVD risk factors (diabetes, high blood pressure, elevated cholesterol) and CVD in fiscal years 1999–2020 within the 6 NAWS regions (California, Southeast, Southwest, East, Midwest, Northwest). Descriptive statistics for continuous variables (e.g., age, income, and years in U.S.) were summarized as means and standard deviations and analyzed using the non-parametric Kruskal-Wallis rank-sum test. Categorical variables (e.g., gender, health insurance, place of birth) were presented as weighted counts and percentages and analyzed using the chi-squared test with Rao and Scott’s second-order correction. These data analyses were performed using R 4.3.0 and SAS Studio.

We presented plots to describe longitudinal trends in the reported prevalence of CVD risk factors and CVD, as well as trends in average summertime temperatures and days above heat thresholds. We mapped the average summertime temperatures from 1999 to 2020 by NAWS region to visualize the regional differences.

We assessed trends in temperature and prevalence of CVD risk factors and CVD with joinpoint regression analysis using Joinpoint Trend Analysis software freely available from the U.S. National Cancer Institute [42]. Joinpoint regression is a method used to identify statistically significant trends in epidemiological time series (annual percent changes) and evaluate non-linearity by allowing ‘joinpoints’ to break the time series into pieces with distinct trends [43, 44].

Finally, we fit multivariate logistic regression models applying a complex survey design to account for the stratified sampling and clustering in NAWS. We estimated odds ratios (ORs) and 95% confidence intervals (CI) with a preselected group of covariates to assess the mutually adjusted associations of each with CVD risk factors and CVD in the total sample. Preselected covariates were: age, gender, benefits from needs-based government programs, hourly work versus piece-rate, mixed and other, years in U.S., migrant status, worker self-report of having health insurance, and region.

## Results

### Descriptive statistics

#### Study participants

A total of 44,388 participants from the NAWS (1999–2020) were available for the analysis, accounting for survey weights in the NAWS public dataset. The number of individuals in each region were: California (*n* = 16,539), Southwest (*n* = 3237), Southeast (*n* = 5346), Northwest (*n* = 6229), Midwest (*n* = 7051), East (*n* = 5986). Descriptive statistics were calculated accounting for the complex survey design that used stratified sampling and clustering.

Table 1 shows sociodemographic, employment, and health characteristics for fiscal years 1999–2020, categorized across the six NAWS regions: California, Southwest, Southeast, Northwest, Midwest, and East.

**Table 1:** Characteristics of farmworkers by region in U.S. National Agricultural Workers Survey (1999–2020); mean (standard deviation) or n (%).

	California	Southwest	Southeast	Northwest	Midwest	East	Total	P*
	*N* = 16,539	*N* = 3237	*N* = 5346	*N* = 6229	*N* = 7051	*N* = 5986	*N* = 44388	0.004
**Demographic characteristics**								
**Age in years**								0.14
	37(13)	41(14)	37(13)	37(13)	37(14)	36(13)	37(13)	
**Gender**								<0.001
Male	12,288(74%)	2529(78%)	3904(73%)	4401(71%)	4906(70%)	4594(77%)	32623(73%)	
Female	4251(26%)	708(22%)	1442(27%)	1827(29%)	2145(30%)	1392(23%)	11,765(27%)	
**Place of Birth is US**								<0.001
U.S. Born	1043(6.3%)	904(28%)	1821(35%)	1018(17%)	3924(56%)	2447(42%)	11,156(25%)	
Not U.S. Born	15,398(94%)	2319(72%)	3385(65%)	5135(83%)	3097(44%)	3442(58%)	32,777(75%)	
Missing	97	14	140	75	30	98	454	
**Adult Primary Language**								<0.001
English	504(3.6%)	509(18%)	1540(33%)	789(15%)	3140(50%)	1907(35%)	8389(22%)	
Spanish	13,013(93%)	2359(82%)	2957(63%)	4404(84%)	3104(49%)	3354(62%)	29,191(76%)	
Indigenous	368(2.6%)	3( < 0.1%)	169(3.6%)	63(1.2%)	40(0.6%)	84(1.6%)	725(1.9%)	
Other	41(0.3%)	2( < 0.1%)	62(1.3%)	7(0.1%)	42(0.7%)	35(0.7%)	189(0.5%)	
Missing	2612	364	619	966	725	607	5893	
**Hispanic**								<0.001
Hispanic	16,075(97%)	2885(89%)	3665(70%)	5523(90%)	3591(51%)	3,969(67%)	35,709(81%)	
Not Hispanic	433(2.6%)	346(11%)	1603(30%)	645(10%)	3403(49%)	1932(33%)	8363(19%)	
Missing	30	5	78	61	56	85	315	
**Number of Years in US**								<0.001
Years (standard deviation)	16(12)	19(13)	12(10)	16(11)	13(11)	11(10)		
Missing	1052	915	1828	1023	3929	2450		
**Highest Grade Completed**								<0.001
No formal schooling	712(4.3%)	134(4.1%)	308(5.8%)	292(4.7%)	107(1.5%)	187(3.1%)	1739(3.9%)	
6th grade or lower	8670(52%)	1240(38%)	1922(36%)	2712(44%)	1761(25%)	2100(35%)	18,406(41%)	
7-9 grade	3817(23%)	680(21%)	1146(21%)	1295(21%)	900(13%)	1007(17%)	8843(20%)	
10-12 grade	2659(16%)	956(30%)	1580(30%)	1287(21%)	2690(38%)	1922(32%)	11,095(25%)	
Education beyond high school	669(4%)	226(7%)	378(7.1%)	632(10%)	1590(23%)	766(13%)	4261(9.6%)	
Other	12( < 0.1%)	0( < 0.1%)	13(0.2%)	10(0.2%)	3( < 0.1%)	5( < 0.1%)	44( < 0.1%)	
**Married**								<0.001
Yes	10,444(63%)	2088(65%)	2841(53%)	3900(63%)	3965(56%)	3086(52%)	26325(59%)	
No	6071(37%)	1147(35%)	2490(47%)	2320(37%)	3084(44%)	2900(48%)	18,012(41%)	
Missing	23	2	15	9	2	1	51	
**Number of Children Under 18 in Household**								<0.001
No children <18	4665(28%)	900(28%)	1273(24%)	1767(28%)	1780(25%)	1091(18%)	27,562(62%)	
1–2 children <18	2012(12%)	369(11%)	481(9%)	936(15%)	646(9.2%)	411(6.9%)	11476(26%)	
3–4 children <18	201(1.2%)	40(1.2%)	54(1%)	76(1.2%)	71(1%)	53(0.9%)	4855(11%)	
Above 4	9660(58%)	1928(60%)	3538(66%)	3450(55%)	4555(65%)	4431(74%)	495(1.1%)	
**Personal Annual Income**								0.20
Dollars (standard deviation)	18,603(10,366)	18,517(10,571)	16,814(10,397)	19,147(10,827)	18,804(12415)	18,108(10083)	18,433(10786)	
Missing	2559	396	991	915	1178	1273	7312	
**Family Income Below Poverty Level**								0.60
Yes	5427(33%)	1117(35%)	1975(37%)	1892(31%)	2156(31%)	1917(32%)	14,485(33%)	
No	11,046(67%)	2112(65%)	3319(63%)	4267(69%)	4853(69%)	4008(68%)	29,606(67%)	
Missing	66	7	52	69	42	61	297	
**Farmworker has Health (Medical) Insurance**								<0.001
Yes	6099(39%)	954(31%)	1236(24%)	1656(28%)	3091(46%)	2017(35%)	15055(36%)	
No	9529(61%)	2127(69%)	3829(76%)	4316(72%)	3665(54%)	3676(65%)	27,144(64%)	
Missing	910	156	280	256	294	293	2189	
**Farmworker’s spouse has Health (Medical) Insurance**								<0.001
Yes	4539(52%)	671(40%)	764(35%)	1139(34%)	1894(54%)	1008(45%)	10,016(46%)	
No	4187(48%)	1028(60%)	1419(65%)	2166(66%)	1607(46%)	1224(55%)	11,630(54%)	
Missing	7813	1538	3163	2923	3550	3755	22741	
**Farmworker’s Employer Provides Health (Medical) Insurance**								<0.001
Yes	13,484(93%)	2229(83%)	3474(81%)	4583(84%)	5084(85%)	3,949(83%)	32,804(87%)	
No	1075(7.4%)	449(17%)	801(19%)	861(16%)	897(15%)	831(17%)	4915(13%)	
Missing	1979	559	1072	784	1070	1206	6669	
**Employment characteristics**								
**Migrant**								0.20
Yes	4118(25%)	737(23%)	1394(26%)	1469(24%)	2053(29%)	1976(33%)	11747(27%)	
No	12407(75%)	2499(77%)	3944(74%)	4743(76%)	4929(71%)	3999(67%)	32522(73%)	
Missing	13	1	8	17	68	11	1180	
**Type of Employer**								<0.001
Grower	10,813(65%)	2770(86%)	4872(91%)	5962(96%)	6977(99%)	5806(97%)	37,200(84%)	
Contractor	5725(35%)	467(14%)	474(8.9%)	267(4.3%)	74(1.1%)	180(3%)	7188(16%)	
**Paid By**								<0.001
By the hour	13,411(81%)	2742(85%)	3990(75%)	5099(82%)	6220(88%)	5100(85%)	36561(82%)	
By the piece-rate	2193(13%)	170(5.3%)	806(15%)	858(14%)	264(3.8%)	414(6.9%)	4706(11%)	
Combination hourly wage/piece	566(3.4%)	12(0.4%)	114(2.1%)	47(0.8%)	50(0.7%)	114(1.9%)	902(2%)	
Salary or other	355(2.1%)	310(9.6%)	427(8%)	216(3.5%)	502(7.1%)	357(6%)	2167(4.9%)	
Missing	14	3	9	8	16	1	51	
**Crop at Time of Interview**								<0.001
Field Crops	625(3.8%)	1035(32%)	1097(21%)	733(12%)	1341(19%)	1648(28%)	6478(15%)	
Fruits & Nuts	9580(58%)	280(8.7%)	955(18%)	3047(49%)	1006(14%)	789(13%)	15657(35%)	
Horticulture	1460(8.8%)	782(24%)	1902(36%)	818(13%)	2769(39%)	1748(29%)	9480(21%)	
Vegetables	4541(27%)	900(28%)	1115(21%)	1357(22%)	1506(21%)	1538(26%)	10957(25%)	
Miscellaneous/Multiple	334(2%)	239(7.4%)	278(5.2%)	274(4.4%)	429(6.1%)	263(4.4%)	1816(4.1%)	
**Hours Worked Last Week at Farm Work**								0.045
Hours (standard deviation)	45(12)	45(15)	43(14)	45(13)	43(14)	45(14)	44(13)	
Missing	406	63	74	255	224	81	1103	
**Farm Work Days in Last 12 Months**								<0.001
Days (standard deviation)	201(97)	198(103)	204(98)	195(102)	167(98)	179(104)	192(100)	
Missing	21	2	49	5	1	23	102	
**Benefits from Contribution-Based Government Programs**								0.008
Yes	4052(25%)	621(19%)	772(14%)	912(15%)	1611(23%)	979(16%)	8948(20%)	
No	12,458(75%)	2614(81%)	4562(86%)	5311(85%)	5420(77%)	4999(84%)	35,363(80%)	
Missing	28	2	13	6	19	8	77	
**Benefits from Needs-Based Government Programs**								<0.001
Yes	7264(44%)	1249(39%)	1925(36%)	2955(47%)	2325(33%)	1796(30%)	17,514(40%)	
No	9246(56%)	1986(61%)	3409(64%)	3267(53%)	4706(67%)	4182(70%)	26797(60%)	
Missing	28	2	13	6	19	8	77	
**Self-reported chronic disease**								
**Cholesterol(2018-2020)**								0.90
Yes	312(13%)	56(17%)	75(14%)	103(12%)	96(15%)	62(12%)	704(13%)	
No	2143(87%)	278(83%)	461(86%)	747(88%)	534(85%)	461(88%)	4624(87%)	
Missing	14,083	2903	4811	5378	6421	5464	39060	
**High Blood Pressure(1999–2020)**								0.90
Yes	1496(9%)	392(12%)	602(11%)	529(8.5%)	733(10%)	529(8.8%)	4280(9.6%)	
No	15,040(91%)	2845(88%)	4745(89%)	5700(92%)	6318(90%)	5458(91%)	40,104(90%)	
Missing	3	0	0	0	0	0	3	
**Diabetes(1999–2020)**								0.14
Yes	844(5.1%)	253(7.8%)	277(5.2%)	338(5.4%)	372(5.3%)	261(4.4%)	2344(5.3%)	
No	15,692(95%)	2984(92%)	5069(95%)	5891(95%)	6679(95%)	5725(96%)	42042(95%)	
Missing	2	0	0	0	0	0	2	
**Heart Disease(1999–2020)**								0.30
Yes	143(0.9%)	48(1.5%)	87(1.6%)	79(1.3%)	82(1.2%)	88(1.5%)	528(1.2%)	
No	16,396(99%)	3188(99%)	5259(98%)	6149(99%)	6969(99%)	5898(99%)	43,859(99%)	

* *P* value from Kruskal-Wallis rank-sum test for complex survey samples; chi-squared test with Rao & Scott’s second-order correction.

##### Sociodemographic characteristics

Overall, the mean age was approximately 37 years old (standard deviation (SD) = 13), with the Southwest having the oldest average age (41 years, SD = 14). The majority of farmworkers were male, comprising 73% of the total population, with similar proportions in all regions. The Midwest had the highest proportion of women among all the regions (30%) with California, the Southeast and the Northwest showing that over a quarter of women work in these regions. Most farmworkers were foreign born (75%), Spanish-speakers (76%) and self-identified as Hispanic (81%). Educational attainment was generally low, with just under half of the workers reporting having completed no more than the 6th grade (41%) or having no formal schooling (4%). More than half of workers (59%) reported being married and a little more than a quarter (26%) reported having 1–2 children under the age of 18 years old.

On average, farmworker’s personal annual income was $18,433, with the highest incomes in the Northwest and the lowest in the Southeast. A third (33%) of workers had family incomes below the poverty level, with the Southeast and Southwest having the highest proportion of these workers (37% and 35%, respectively). Only 36% of farmworkers reported being individually enrolled in medical insurance, with the Southeast reporting the lowest proportion of individuals with insurance (24%). Similarly, more than half of workers who reported being married (54%) reported that their spouse lacked health insurance coverage. The Midwest had the highest rate of individually insured workers (46%), while the Midwest (54%) and California (52%) showed the highest spousal insurance coverage.

##### Employment characteristics

Migrant farmworkers accounted for a little more than a quarter (27%) of all workers with the East region having the highest percentage and the Southwest region the lowest. The majority of workers reported working with a grower (84%) and only 16% worked for a contractor. Most farmworkers (82%) were paid hourly, with the Midwest having the highest proportion of hourly-paid workers (88%). The Southeast had the highest proportion of farmworkers paid by piece rate, with 15% receiving this form of compensation. Meanwhile, the Southwest had the highest proportion of salaried workers (10%) compared to just 2.1% in California. Regarding crops, 35% of farmworkers worked with fruits and nuts, with California leading at 58%, followed by 27% in vegetables. The Southeast (36%) and Midwest (39%) reported the highest involvement in horticulture.

On average, across all regions, workers reported working 44 h in the week prior to the interview. Over the past 12 months, they worked an average of 192 days on crop work. Among the regions, California and the Southeast had the highest number of workdays, while the Midwest reported the fewest. In terms of federal benefits, 20% of workers accessed contribution-based programs like unemployment insurance or Social Security, while 40% utilized need-based programs such as Medicaid or the SNAP. California had the highest reliance on contribution-based programs, while the Northwest had the highest proportion of participants in the needs-based program usage.

##### Prevalence of CVD risk factors and CVD

Among all the CVD risk factors, elevated cholesterol had the highest prevalence across all regions (12–17%), with farmworkers in the Southwest showing the highest prevalence; cholesterol was self-reported only from 2018 to 2020 (*n* = 4624) in NAWS. High blood pressure prevalence ranged from 9 to 12%, with the highest again in the Southwest (12%). Diabetes was more prevalent in the Southwest (8%), while the other regions showed prevalence in the range of 4% and 5%. For CVD, California had the lowest self-reported prevalence at 1%, while the Southeast and East regions had the highest at 2%. The other regions (Southeast, Midwest, Northwest) had a similar prevalence around 1%.

### Heat metrics in the six regions of the National Agricultural Workers Survey

Table 2 presents the heat exposure metrics, which represent region-level averages based on the survey-weighted responses in the farmworker dataset. Because the NAWS uses a complex survey design with clustering, stratification, and weights, even simple summary estimates, like average temperature exposure, require proper design-adjusted estimation. The 95% confidence intervals (CIs) reflect the precision of those survey-weighted means. Significant differences were observed across regions for all variables, as indicated by *p* values < 0.001 derived from the Kruskal-Wallis rank-sum test for complex survey samples.

**Table 2:** Heat metrics [mean (standard deviation) (SD)]in the six regions of the National Agricultural Worker Survey (NAWS) (1999–2020). 95% confidence intervals (CI) reflect precision accounting for the NAWS weighted survey structure. Extreme heat days and temperatures are presented as means (standard deviation (SD)).

	California, *N* = 16,539				Southwest, *N* = 3237				Southeast, *N* = 5346				Northwest, *N* = 6229				Midwest, *N* = 7051				East, *N* = 5986				P*		All, *N* = 44388	
	Mean (SD)		95% CI		Mean (SD)		95% CI		Mean (SD)		95% CI		Mean (SD)		95% CI		Mean (SD)		95% CI		Mean (SD)		95% CI				Mean (SD)	
Extreme Heat Days **																												
Temperatures > 32.2 °C (days)	54.0 (7.0)		(53.3, 55.4)		101.0 (16.0)		(97.7, 103.6)		60.0 (23.0)		(54.6, 65.2)		17.3 (3.6)		(16.8, 17.8)		30.0 (11.0)		(28, 32.5)		15.0 (8.0)		(13.2, 16.5)		<0.001		44.0 (26.0)	
Temperatures > 35.0 °C (days)	35.0 (5.9)		(34.1, 36)		58.0 (20.0)		(54.7, 61.6)		19.0 (16.0)		(15.8, 21.5)		6.5 (1.9)		(6.2, 6.7)		13.0 (9.0)		(11.6, 14.7)		3.2 (3.1)		(2.7, 3.7)		<0.001		23.0 (18.0)	
Temperatures > 37.8 °C (days)	19.4 (4.6)		(18.6, 20.2)		24.0 (15.0)		(21.7, 27.2)		4.4 (5.4)		(3.5, 5.2)		1.5 (0.7)		(1.5, 1.6)		5.0 (5.0)		(4.2, 5.9)		0.6 (1.0)		(0.4, 0.7)		<0.001		11.0 (11.0)	
Temperatures > 40.6 °C (days)	8.4 (2.6)		(7.9, 8.8)		5.7 (6.1)		(4.6, 6.8)		0.7 (1.0)		(0.5, 0.8)		0.2 (0.1)		(0.2, 0.2)		1.5 (2.5)		(1.1, 1.9)		0.1 (0.2)		(0, 0.1)		<0.001		3.9 (4.5)	
Spring and Summer Temperatures***																												
Average spring temperature (°C)		13.6 (1.9)		(13.4, 13.6)		15.4 (1.3)		(15.3, 15.6)		18.3 (1.5)		(18.2, 18.4)		6.9 (1.6)		(6.9, 7.1)		9.1 (2.5)		(8.9, 9.3)		10.0 (1.7)		(9.9, 10.1)		<0.001		12.2 (6.0)
Average summer temperature (°C)		23.6 (1.3)		(23.4, 23.7)		25.9 (1.1)		(25.8, 26.0)		26.8 (1.0)		(26.7, 26.8)		18.8 (1.1)		(18.7, 18.9)		21.8 (1.5)		(21.7, 21.9)		21.7 (1.1)		(21.6, 21.7)		<0.001		22.9 (4.3)

* *p* value based on Kruskal-Wallis rank-sum test for complex survey samples.

**Data from National Environmental Public Health Tracking Network (U.S. Centers for Disease Control and Prevention (CDC)) https://ephtracking.cdc.gov/DataExplorer/.

***Data from National Centers for Environmental Information (U.S. National Oceanic and Atmospheric Administration (NOAA)) https://www.ncei.noaa.gov/access/monitoring/climate-at-a-glance/.

Regionally, California and Southwest showed the highest frequency of extreme heat days. Notably, for days exceeding 40.6 °C, California reports the highest average, with 8.4 days (standard deviation (SD) = 2.6). The East (mean = 0.1 days, SD = 0.2) and Northwest (mean = 0.2 days, SD = 0.1) had the lowest number of days with heat exceeding that threshold.

Seasonal temperature patterns varied significantly across regions (*p* < 0.001) (Table 2). During spring, the Southeast, Southwest and California recorded the highest average temperatures, compared to the East and Midwest, and Northwest. Similar regional patterns were observed with the Southeast having the highest average summer temperature followed by the Southwest and California. The Northwest had the lowest summer temperatures.

**Fig. 1:**
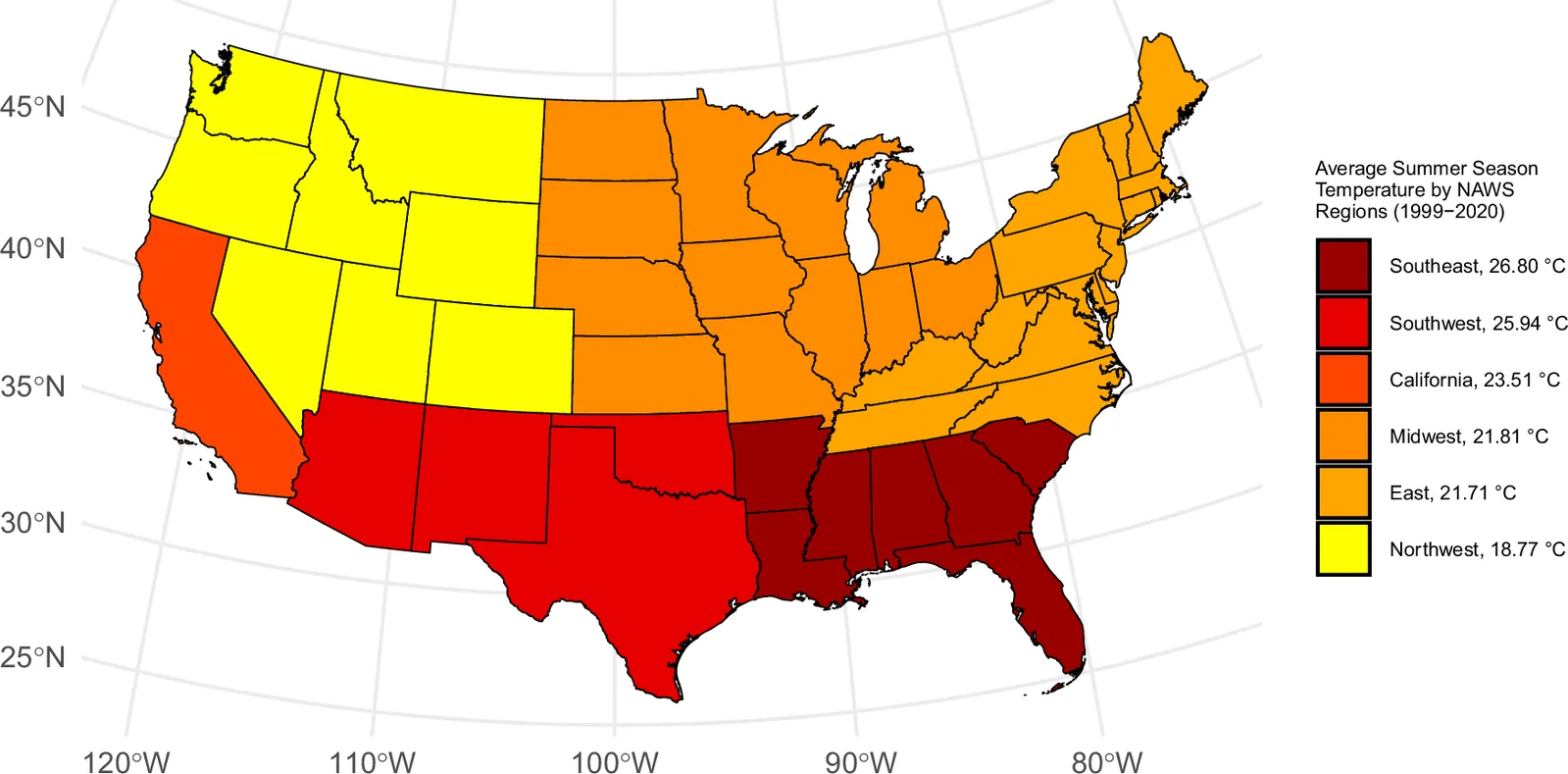
Average summertime season temperatures in the six regions of the U.S. National Agricultural Workers Survey (NAWS), 1999–2020.

**Fig. 2:**
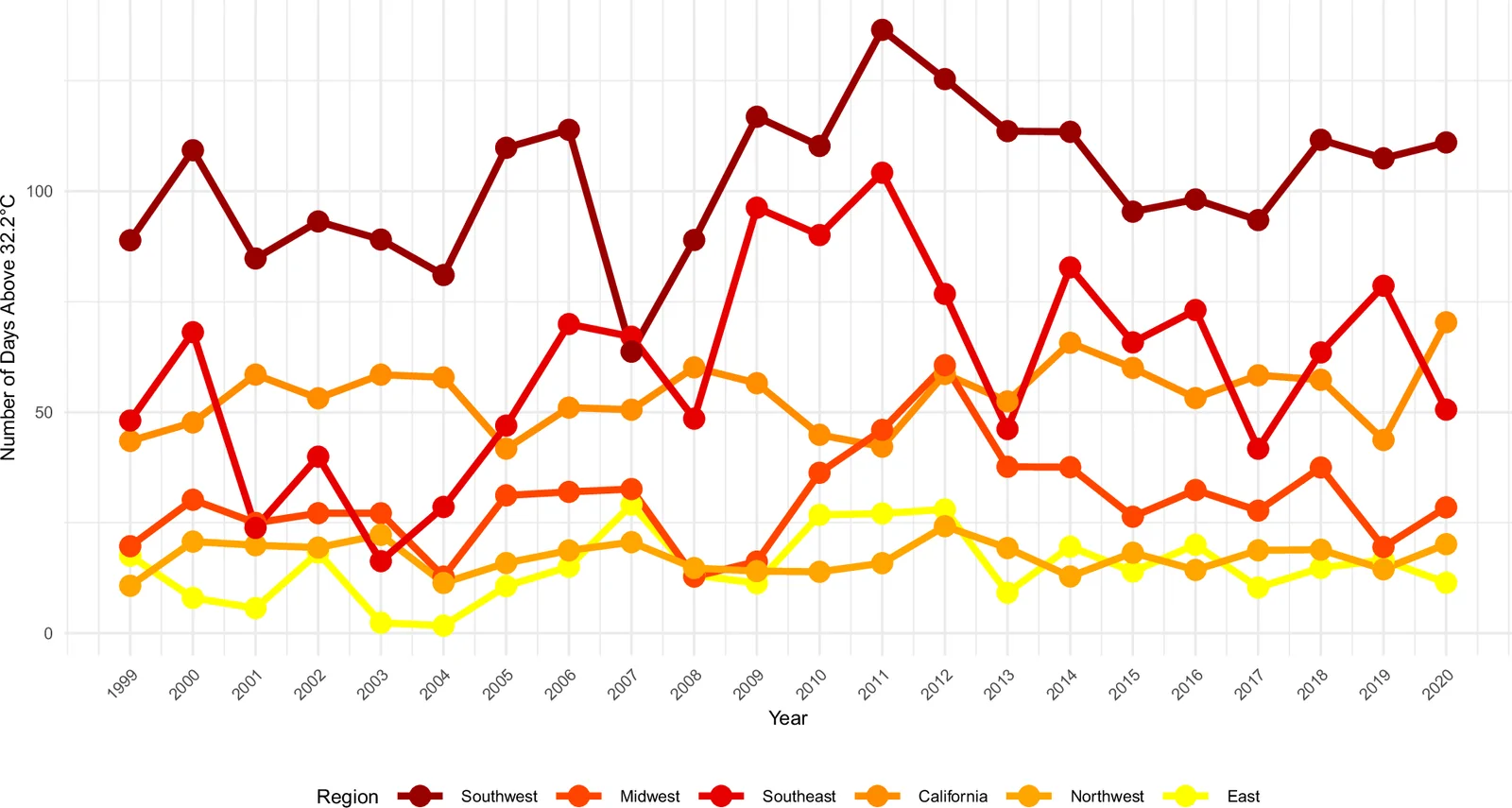
Region-specific time series of annual days with temperatures above 32.2 °C threshold for the U.S. National Agricultural Workers Survey, 1990–2020.

Figure 1 illustrates the regional differences in average summertime temperatures, with the southeast and southwest being the warmest of the six NAWS regions. Figure 2 shows region-specific time series of the days in the year above the 32.2 °C threshold. Considerably annual variability in the numbers of days is seen, with again the southwest and southeast regions overall having the highest counts of extreme heat days in the time period.

### Join point time trends in disease prevalence and temperature metrics

**Fig. 3:**
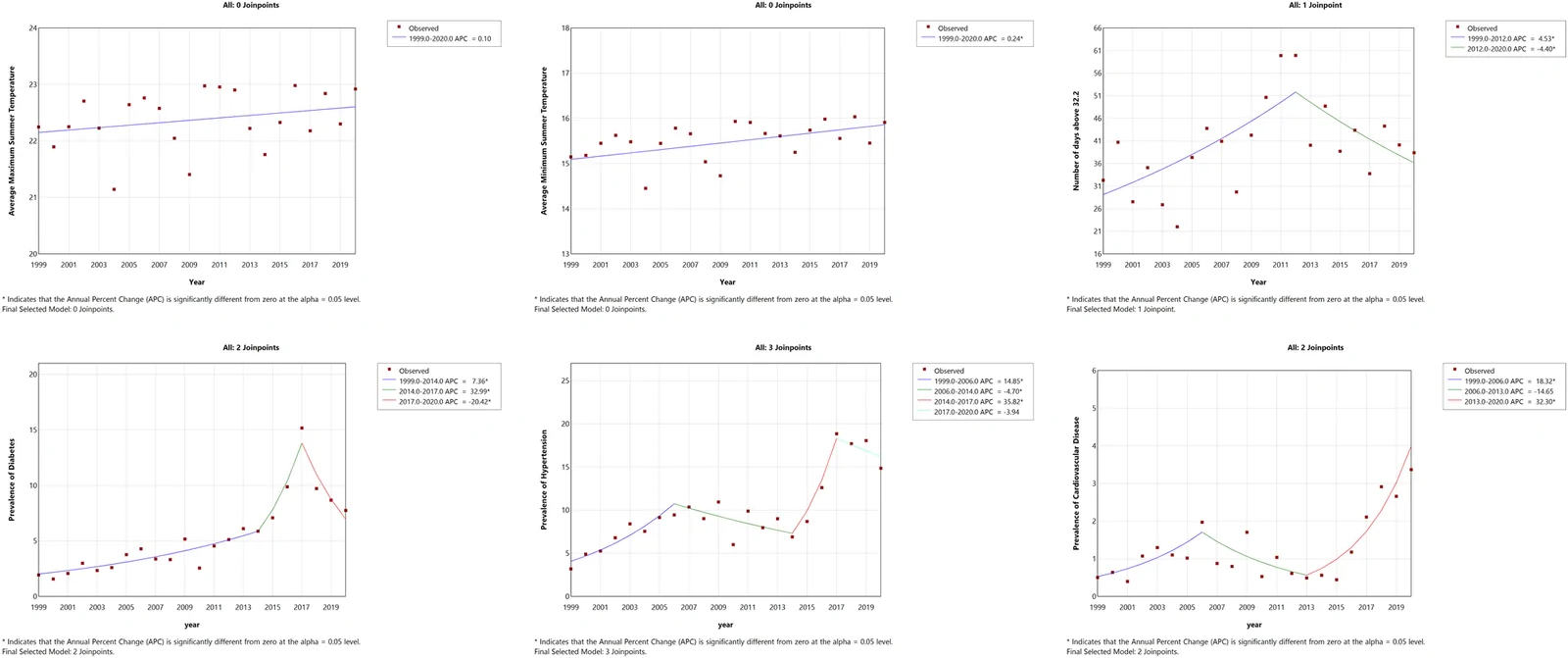
Time trends and annual percent change in chronic disease prevalence and warm temperature exposures, 1990–2020, using joinpoint regression. The top panel shows trends in summertime minimum and maximum temperatures and days above a heat threshold (32.2 °C). The bottom panel shows prevalence of high blood pressure, diabetes and cardiovascular disease as reported by respondents to the U.S. National Agricultural Workers Survey.

Join point regressions for the temperature metrics and reported prevalence of diabetes, high blood pressure, and CVD are shown in Fig. 3. The top panel of the figure shows a gradual monotonic increase in maximum and minimum summer temperatures over the period, with only minimum summer temperatures displaying a significant annual percent change of 0.24%. The overall average annual number of days over the 32.2 °C threshold increases until about 2012 and then decreases after that date, with about a 4.5% significant change in each direction. Diabetes prevalence increases significantly over the period, until dropping after 2017, but still remains higher after 2017 than all prevalences reported prior to 2015. High blood pressure prevalence shows a more complex, non-linear pattern, with prevalences generally higher in the later part of the period, with a non-significant reduction in annual percent change after 2017. CVD also showed a nonlinear trend, with a period of non-significant decrease between 2006 and 2013 but a steep and significant rise of 32.3% annual percent change from 2013 to 2020.

These increases in disease prevalence in the NAWS sample are reflective of the overall increasing age of hired crop farmworkers (Supplementary Fig. 1), with average age of respondents under 34 years in 1999 and over 42 years in 2020.

### Associations of farmworker characteristics with CVD and CVD risk factors

Table 3 shows that increasing age was consistently positively and significantly associated with experiencing high blood pressure, diabetes, elevated cholesterol and CVD, as expected. Similarly, workers reporting having health insurance was associated with prevalence of these conditions, except for cholesterol and heart disease where the CI was wide. This association is expected given that the survey question is based on being informed by a provider of a health condition which means the individual had some access to health care services. Odds of high blood pressure and diabetes were significantly associated with having needs-based government benefits (e.g., Medicaid, WIC, SNAP), suggesting that individuals with these chronic conditions have other social and health-related challenges. Other worker characteristics had inconsistent associations with the outcomes assessed in this study.

**Table 3:** Farmworker characteristics and odds of cardiovascular risk factors and cardiovascular disease in the National Agricultural Workers Survey (NAWS) for 1999–2020; adjusted odds ratios and 95% confidence intervals (CIs) from survey weighted multivariate logistic regression models.

	Heart disease	Hypertension	Diabetes	Cholesterol (2018–20)
**Covariate**	*n* = 31,173	*n* = 31,170	*n* = 31,173	*n* = 3783
	Odds Ratio (95% CI)	Odds Ratio (95% CI)	Odds Ratio (95% CI)	Odds Ratio (95% CI)
Age	1.05 (1.02, 1.07)	1.07 (1.06, 1.08)	1.06 (1.05, 1.07)	1.04 (1.02, 1.06)
Gender (Woman as referent)	0.79 (0.54, 1.17)	0.68 (0.56, 0.83)	0.56 (0.46, 0.68)	1.21 (0.72, 2.05)
Needs-based government benefits	1.59 (0.72, 3.50)	1.24 (1.06, 1.45)	1.50 (1.20, 1.88)	0.79 (0.49, 1.27)
Type of pay (hourly as referent)				
Piece-rate	0.51 (0.22, 1.20)	0.70 (0.50, 0.97)	0.63 (0.48, 0.83)	0.51 (0.13, 2.00)
Mixed hourly and piece-rate	1.28 (0.68, 2.15)	0.88 (0.54, 1.45)	0.62 (0.31, 0.83)	0.40 (0.13, 1.21)
Other	2.32 (0.99, 4.17)	1.11 (0.74, 1.65)	1.06 (0.68, 1.23)	1.76 (0.79, 3.92)
Years in U.S.	1.02 (1.00, 1.04)	1.01 (1.00, 1.02)	1.02 (1.01, 1.03)	1.00 (0.98, 1.03)
Migrant versus not migrant	1.11 (0.68, 1.80)	0.76 (0.62, 0.92)	0.83 (0.62, 1.12)	0.81 (0.51, 1.03)
Self report of worker having health insurance	1.35 (0.76, 2.42)	1.37 (1.13, 1.66)	1.51 (1.14, 1.98)	1.57 (0.99, 2.48)
NAWS region (California as referent)				
East	1.59 (0.72, 3.50)	0.94 (0.69, 1.28)	1.48 (0.97, 2.24)	0.81 (0.44, 1.51)
Midwest	0.51 (0.22, 1.20)	0.86 (0.63, 1.16)	1.59 (1.08, 2.34)	1.40 (0.47, 4.12)
Northwest	1.34 (0.68, 2.61)	0.92 (0.72, 1.17)	1.15 (0.89, 1.49)	1.00 (0.55, 1.84)
Southeast	1.78 (0.90, 3.54)	0.95 (0.71, 1.29)	0.99 (0.70, 1.41)	1.67 (0.66, 4.24)
Southwest	0.65 (0.30, 1.40)	0.93 (0.71, 1.20)	1.37 (1.01, 1.86)	1.03 (0.56, 1.89)

## Discussion

To our knowledge, this is the first study to assess national level data with 21 years of data on hot temperature exposures and CVD outcomes with NAWS. Our results contribute to the body of knowledge about farmworkers, their health status and the potential vulnerability to occupational heat stress of farmworkers [15, 21, 45–50]. Our findings reveal trends in chronic disease prevalence that potentially enhance susceptibility of these workers to heat exposure and impact the safety, health and wellbeing of a workforce essential to providing healthy food and other beneficial crops to the U.S.

The study results highlight the social vulnerability of farmworkers. Notably, more than a third reported family incomes below the poverty level, and most workers reported lacking health insurance (64%). Farmworkers are a population already at increased risk for adverse health outcomes due to socioeconomic and structural barriers. Poverty, legal precarity, and lack of health insurance and inadequate healthcare access among farmworkers exacerbates their susceptibility to occupational hazards, including heat exposure.

Generally, the literature on CVD factors among farmworkers is limited and results from studies present some inconsistencies. The prevalence of self-reported high blood pressure (9.6%) and elevated cholesterol (13%) found in our study is lower than findings by Matias et al. (2022) using clinical data from three studies (population-based, pilot, and a cluster-randomized controlled trial) with 1300 Hispanic farmworkers in California. Matias and colleagues reported that almost half the sample had hypertension (42.0–45.5%), and approximately a quarter of participants had high total cholesterol (23.7–25.8%) [45]. The 2013 Behavioral Risk Factor Surveillance System (BRFSS) survey of workers in farming, fishing and forestry reported prevalences (adjusted by age, sex, race-ethnicity and education) and 95% CIs for having been told by a doctor they have various chronic conditions. These were 21.7% (13.3–33.3%) for high blood pressure; 27.1% (17.4–39.7%) for elevated cholesterol; and 4.2% (1.9–9.0%) for diabetes [46]. Results in our NAWS sample were 9.6% for high blood pressure, 13.0% for elevated cholesterol and 5.3% for diabetes over the time period. Differences across these surveys could relate to the fact that: the NAWS sampling strategy is employer based and thus may capture a higher proportion of seasonal/migrant farmworkers; the BRFSS category includes forestry and fishing, and self-report based on diagnoses may underestimate the true prevalence of disease. Indeed, the Farm Worker Family Health Program and research studies in Florida with clinical data from 728 agricultural workers, indicated a higher proportion of elevated blood pressure (70% Florida sample, 67% Georgia sample) [15] than what we found in our study. A meta-analysis of the prevalence of CVD risk factors among Hispanic migrant and seasonal farmworkers showed a wide range of prevalence rates of diabetes mellitus for females (2%-57%) and males (1%-63%) [47]. The inconsistency in the prior studies and our findings demonstrate the need to conduct prospective studies on cardiovascular health in farmworkers, but the existence of these chronic health conditions in an active working population prone to experience heat stress supports preventive intervention. Indeed, it is worth re-emphasizing the strong likelihood of underreporting in the dataset, since answering yes to any of the symptoms depended on whether the farmworker had seen a health care provider. Other researchers have highlighted regional variation in health conditions among respondents to the NAWS, as well as factors which affect access to health care [48, 49], important considerations in tailoring interventions to local conditions.

Currently, only five states have heat standards for outdoor workers (California, Colorado, Oregon, Washington, and Maryland) with the enforcement of heat-illness prevention standards and measures prompted at various heat thresholds [6, 31]. Yet, recent evidence suggests that measures including provision of shade, frequent resting, and hydration not only protects the health of farmworkers but also increases productivity, a win-win for both employers and farmworkers [50].

In 2023, the U.S. Congress Appropriations Committee charged the Department of Labor with developing heat-related illness and heat-related training questions to include in the next administration of the NAWS, given the importance of the issue [51]. At a May 2025 conference, Dr. Andrew Padovani, director of the NAWS, presented results from qualitative interviews of farmworkers in Texas and California, conducted by NAWS staff in 2024, that will inform the development of these questions [52]. The interviews revealed numerous concerns by farmworkers, including disincentives to rest and maintain hydration, consistent with work by Hyland and colleagues [53], suggesting that the new information provided by NAWS when these questions are included may be of great value for informing Federal policy.

The contributions of the present research must be considered in the context of its limitations. A primary limitation is the repeated cross-sectional study design and the use of self-reported health outcome data. The present analysis evaluates individual health conditions reported by farmworker respondents, but up to 4.1% of the respondents (in the Southwest region, consistent with results of Soto et al. [48]) report two or more conditions (see Supplemental Information) which could even further increase risk of HRI. Selection bias is an inherent limitation in NAWS as for example in years 2019-2022, 52% of the randomly selected eligible growers (farms) agreed to participate with the survey and in these farms, most of the workers sampled (92%) agreed to be interviewed (personal communication NAWS, Sept 30, 2024). Thus, it may be that farmworkers with precarious work conditions and highest risk for heat exposure and disease were not included in the survey. Misclassification of self-reported disease outcomes is also plausible. We reiterate here that the NAWS does not interview H2A workers, now a significant segment of the agricultural labor force in the U.S., and so characteristics of those workers may differ from those seen in the NAWS sample.

Aggregated measures from temperatures at the regional level may not accurately reflect the true maximum air temperature and heat index in all locations and may differ from other temperature data sources or defined temperature measures. Additionally, we acknowledge that humidity along with temperature can contribute importantly to heat strain, particularly in regions such as the southeast. However, outdoor air temperature data are widely used in temperature epidemiology [54], and given that farm work occurs outdoors, the exposure estimates are likely valid for the purpose of describing regional and temporal temperature characteristics over the time period and further current knowledge on the potential vulnerabilities to hot temperatures in agricultural workers. We made use of the NAWS, a nationally-representative dataset with information on CVD risk factors and CVD for farmworkers, including seasonal and migrant farmworkers.

These results underscore the need to implement a public health surveillance system of heat stress for farmworkers and outdoor workers in general. The study has policy implications in light of the OSHA’s preliminary regulatory framework on worker safety and health, enacted in April 2024, and the acknowledged risks that heat stress poses to farmworkers and other workers exposed to high temperatures. As noted in a recent review by El Khayat and colleagues [55], many of the risk factors for heat-related illness have to do with work conditions. In places where piece-rate work; lack of workplace protections; higher frequency of hot weather, and high prevalence of predisposing health conditions converge (e.g., the Southeast) risks for HRI may be increased, relative to other areas represented by the NAWS. Enhanced understanding of worker’s heat exposure, and physical exertion in outdoor workers, is vital to inform occupational safety and health policies with a focus on cardiovascular health, specifically for outdoor workers like farmworkers.

## Conclusion

This study contributes to the growing body of evidence on concerns about heat exposure and farmworker health, highlighting regional differences in CVD risk and the social vulnerabilities that influence these outcomes. Comprehensive public health strategies are needed to mitigate the effects of extreme heat and protect farmworkers’ health, especially as climate change increases the frequency and intensity of heat events.

## Supplementary information

Supplementary information

Supplemental Figure 1

## Data Availability

The temperature data analyzed in this paper are from public sources as noted in methods. The National Agricultural Workers Survey data were obtained through a data use agreement with the Department of Labor. Codes used to model and clean data are available from the corresponding author on reasonable request.
