# Supplementary material for: Heat exposure and cardiovascular risk factors among migrant and seasonal farmworkers in the United States: data from the National Agricultural Workers Survey (1999–2020)
Source: J Expo Sci Environ Epidemiol. 2026 May 30;36(6):1032–44. doi: 10.1038/s41370-026-00919-x (PMC13727674; doi:10.1038/s41370-026-00919-x)
Supplement: Supplementary file 1 [file 41370_2026_919_MOESM2_ESM.pdf]

Supplemental Figure 1. Self-reported age among respondents to the National Agricultural Workers Survey, 1999-2020.

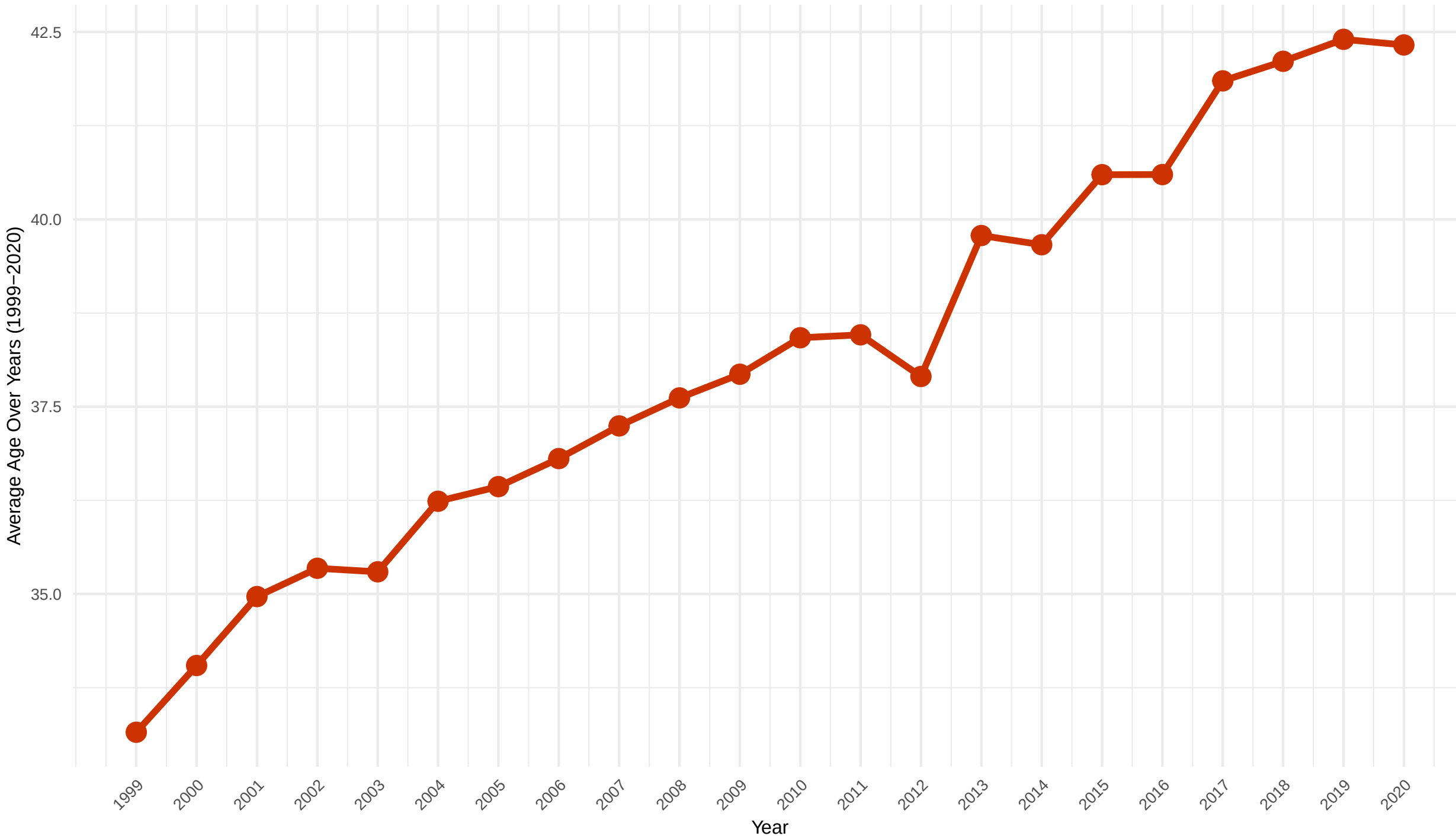

Average Age Over Years (1999–2020)
42.5
40.0
37.5
35.0
1999
2000
2001
2002
2003
2004
2005
2006
2007
2008
2009
2010
2011
2012
2013
2014
2015
2016
2017
2018
2019
2020
Year
