# Supplementary material for: Heat exposure and cardiovascular risk factors among migrant and seasonal farmworkers in the United States: data from the National Agricultural Workers Survey (1999–2020)
Source: J Expo Sci Environ Epidemiol. 2026 May 30;36(6):1032–44. doi: 10.1038/s41370-026-00919-x (PMC13727674; doi:10.1038/s41370-026-00919-x)
Supplement: Supplementary file 2 [file 41370_2026_919_MOESM1_ESM.docx]

**Supplemental Information on Reported Outcomes**

**Missing Data**

In our dataset, NH02 (diabetes), NH03 (high blood pressure), and NH05 (heart disease) are nearly complete (missing <0.01%). In contrast, NH12 (high cholesterol) was only asked among ~12% of participants, leaving ~88% missing. For the main analyses, we thus focused on the frequency of outcomes using NH02, NH03, and NH05. Approximately 88% of participants answered three items (NH02, NH03, NH05), 12% answered four items (including NH12), and only ~0.01% answered two items.

**Overall frequency of health outcomes**
 Weighted descriptive results for NH02, NH03, and NH05 among all NAWS farmworkers showed:

- 0 outcomes: ~38,528 participants (86.8%, 95% CI: 85.9–87.7)
- 1 outcome: ~4,672 participants (10.5%, 95% CI: 9.8–11.2)
- 2 outcomes: ~1,081 participants (2.4%, 95% CI: 2.1–2.9)
- 3 outcomes: ~105 participants (0.24%, 95% CI: 0.16–0.36)

The majority of participants (nearly 87%) reported no outcomes, ~10% reported one outcome, ~2% reported two outcomes, and only a very small fraction (<1%) reported three outcomes.

**Regional distribution of health outcomes**

Weighted estimates by region were as follows:

- **California (N ≈ 16,539):** 87.4% with 0 outcomes (≈14,446), 10.5% with 1 outcome (≈1,729), 2.0% with 2 outcomes (≈338), 0.2% with 3 outcomes (≈26).
- **East (N ≈ 5,986):** 88.3% with 0 outcomes (≈5,285), 9.3% with 1 outcome (≈557), 1.9% with 2 outcomes (≈112), 0.5% with 3 outcomes (≈32).
- **Midwest (N ≈ 7,051):** 86.0% with 0 outcomes (≈6,063), 11.2% with 1 outcome (≈793), 2.7% with 2 outcomes (≈192), <0.1% with 3 outcomes (≈3).
- **Northwest (N ≈ 6,229):** 87.7% with 0 outcomes (≈5,465), 9.6% with 1 outcome (≈597), 2.4% with 2 outcomes (≈149), 0.3% with 3 outcomes (≈17).
- **Southeast (N ≈ 5,346):** 85.2% with 0 outcomes (≈4,557), 11.7% with 1 outcome (≈623), 2.9% with 2 outcomes (≈156), 0.2% with 3 outcomes (≈11).
- **Southwest (N ≈ 3,237):** 83.8% with 0 outcomes (≈2,712), 11.5% with 1 outcome (≈373), 4.1% with 2 outcomes (≈134), 0.5% with 3 outcomes (≈17).

Across regions, the majority of participants consistently reported no outcomes (83–88%), ~9–12% reported one outcome, ~2–4% reported two outcomes, and <1% reported three outcomes. The Southwest region had the highest relative proportion of participants with two or more outcomes.
